# Supplementary material for: Differential activation of Fyn kinase distinguishes saturated and unsaturated fats in mouse macrophages
Source: Oncotarget. 2017 Sep 21;8(49):86634–45. doi: 10.18632/oncotarget.21258 (PMC5689713; doi:10.18632/oncotarget.21258)
Supplement: Supplementary file 1 [file oncotarget-08-86634-s001.pdf]

Differential activation of Fyn kinase distinguishes saturated and unsaturated fats in mouse macrophages

SUPPLEMENTARY MATERIALS

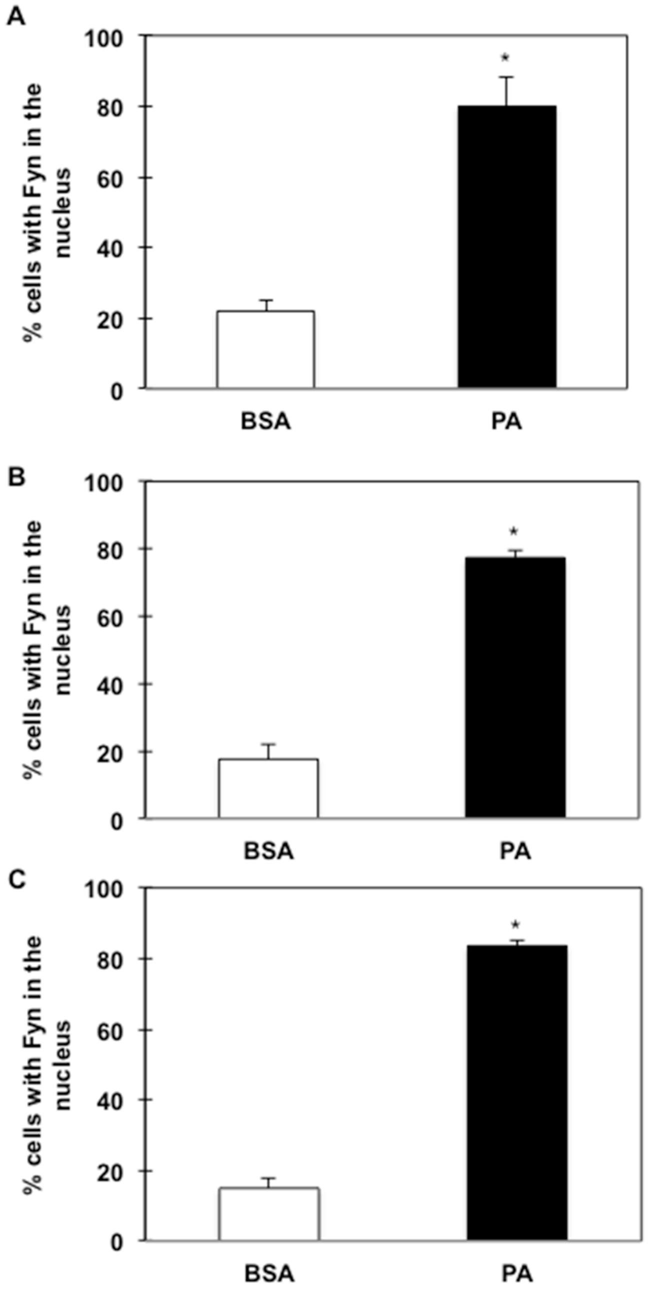

Supplementary Figure 1: (A) RAW264.7 cells **(B)** BMDMs from wild type mice and **(C)** J774 cells were treated with BSA alone (open bars) or 150 µM palmitate (PA; dark bars) (PA:BSA=2) for 10 minutes. Fyn subcellular localization was assessed by immunofluorescence. Cells showing Fyn signal in the nucleus were counted as positive, (n= 7 experiments), * *p* (PA *vs.* BSA)<0.05.

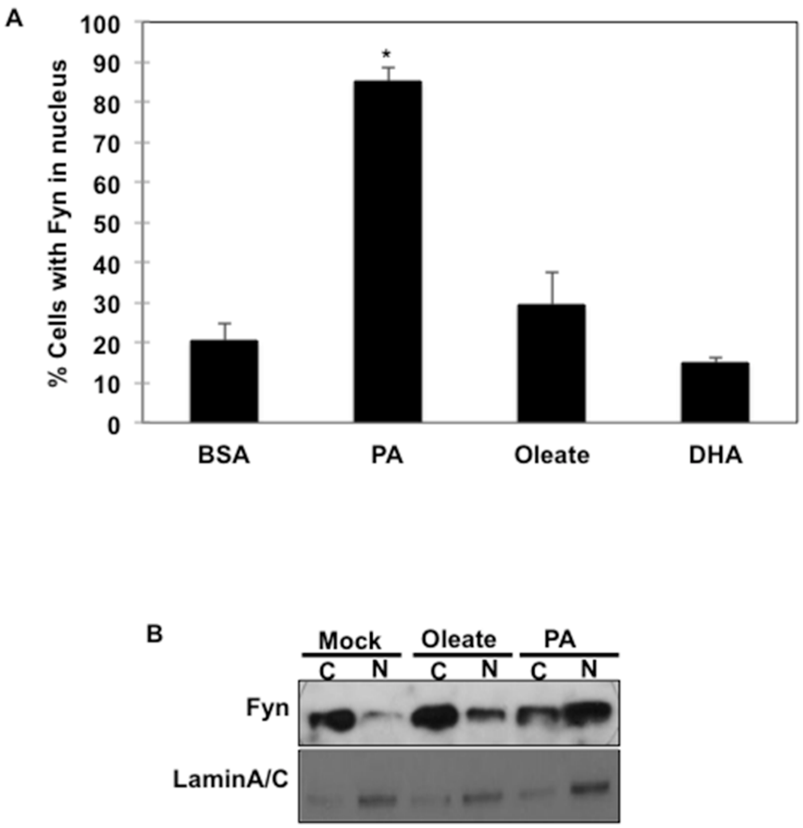

Supplementary Figure 2: (A) RAW264.7 cells were treated with 150 µM palmitate (PA), oleate or DHA (FA:BSA= 2) for 10 minutes. Fyn subcellular localization was assessed by immunofluorescence. Cells showing Fyn signal in the nucleus were counted as positive, (n=5 experiments), * *p* (FA *vs*. BSA)<0.05. **(B)** RAW264.7 cells were treated with 150 µM palmitate (PA) or oleate for 10 minutes. Cytoplasmic (C) and nuclear (N) extracts were prepared and Fyn protein expression was assessed by immunoblotting. LaminAC was used as nuclear loading control.

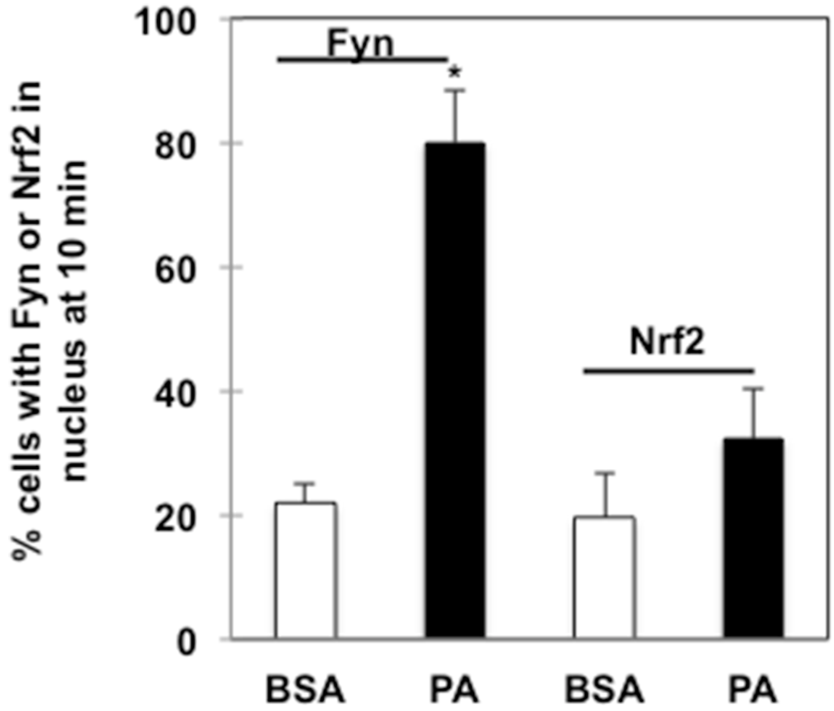

Supplementary Figure 3: RAW264.7 macrophages were treated with 150 µM palmitate (PA) for 10 minutes. (PA:BSA=2). Fyn and Nrf2 subcellular localization was assessed by immunofluorescence. Cells with Fyn or Nrf2 signal in the nucleus were counted as positive, (n=4 experiments), *p<0.05 (PA *vs*. BSA).

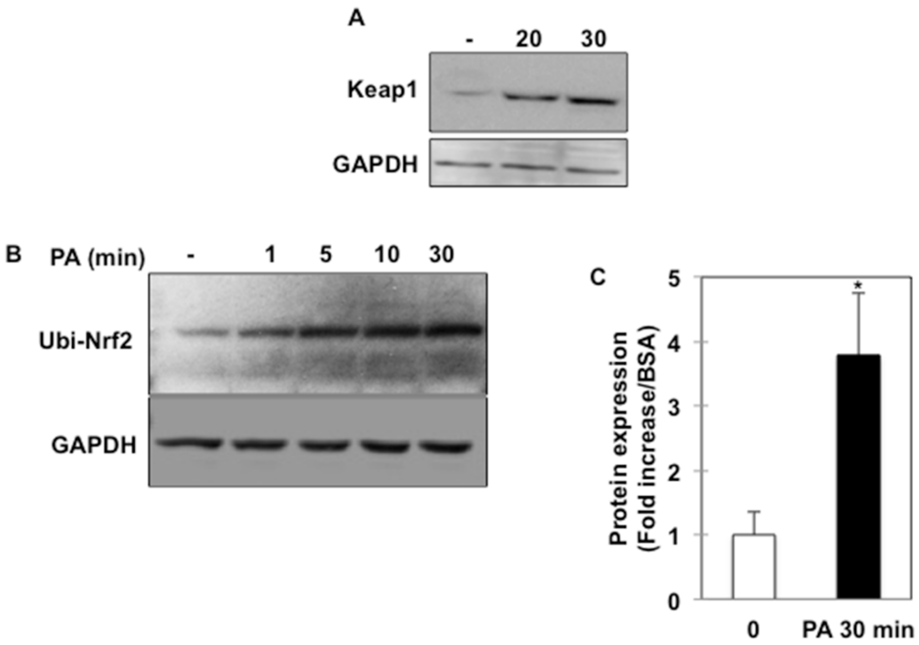

Supplementary Figure 4: Palmitate increases Nrf2 ubiquitination **(A)** Cells were incubated with BSA (-) or 150 µM palmitate (PA) for the indicated times. Keap1 protein expression was assessed by immunoblotting. **(B)** Cells were incubated with BSA (-) or 150 µM palmitate (PA) for the indicated times. Ubiquitinated Nrf2 (Ubi-Nrf2) expression levels were assessed by immunoblotting using the Nrf2-C-20 antibody from Santa Cruz (molecular weights of Nrf2 and poly-ubiquitinated Nrf2 are 57 and 100KDa, respectively (according to the manufacturer's datasheet)). **(C)** Signal quantification of ubiquitinated Nrf2 expression. Ubi-Nrf2 signal was corrected by the GAPDH signal. The graph represents the fold increase of the Ubi-Nrf2 signal after a 30 min PA incubation compared to BSA condition (0), *p<0.05.

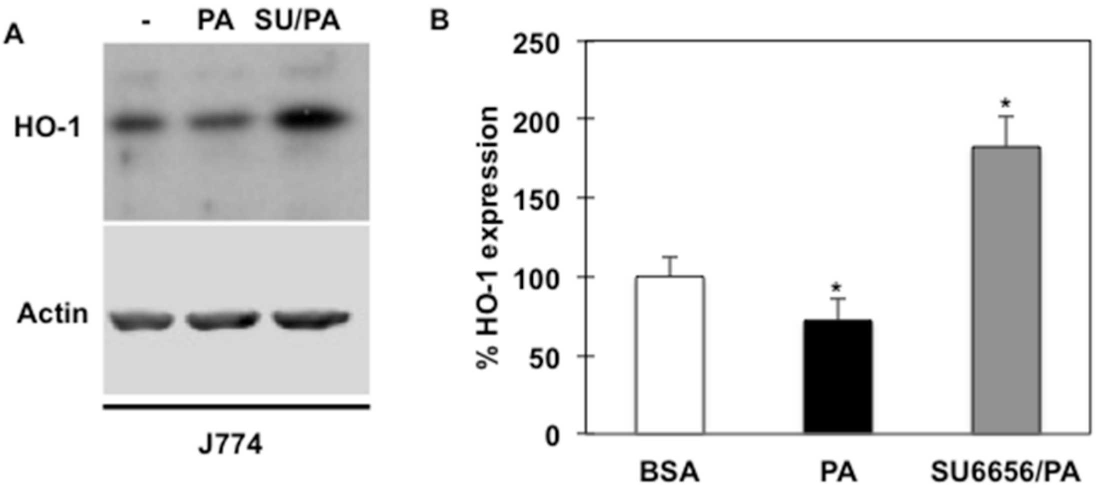

Supplementary Figure 5: (A) HO-1 protein expression in J774 macrophages in response to 150 mM palmitate (PA) or 5 mM SU6656 and 150 mM palmitate (PA) (SU/PA) (n=3 experiments). **(B)** Signal quantification of HO-1 protein expression in RAW264.7 cells corrected by actin and compared to BSA condition, * $p<0.05$ (n=3).
